# Evaluation of Strength Development in Concrete with Ground Granulated Blast Furnace Slag Using Apparent Activation Energy

**DOI:** 10.3390/ma13020442

**Published:** 2020-01-17

**Authors:** Hyun-Min Yang, Seung-Jun Kwon, Nosang Vincent Myung, Jitendra Kumar Singh, Han-Seung Lee, Soumen Mandal

**Affiliations:** 1 Innovative Durable Building and Infrastructure Research Center, Department of Architectural Engineering, Hanyang University, 1271 Sa 3-dong, Sangnok-gu, Ansan 15588, Koreajk200386@hanyang.ac.kr (J.K.S.); 2 Department of Chemical and Environmental Engineering, University of California-Riverside, Riverside, CA 92521, USA; myung@engr.ucr.edu; 3 Department of Civil and Environmental Engineering, Hannam University, Daejeon 34430, Korea; jjuni98@hannam.ac.kr; 4 Department of Architectural Engineering, Hanyang University, 1271 Sa 3-dong, Sangnok-gu, Ansan 15588, Korea; 5 Intelligent Construction Automation Center, Kyungpook National University, 80, Daehak-ro, Buk-gu, Daegu 41566, Korea

**Keywords:** compressive strength, concrete, ground granulated blast furnace slag, apparent activation energy, equivalent age

## Abstract

Ground granulated blast furnace slag (GGBFS) conventionally has been incorporated with ordinary Portland cement (OPC) owing to reduce the environmental load and enhance the engineering performance. Concrete with GGBFS shows different strength development of normal concrete, but sensitive, to exterior condition. Thus, a precise strength evaluation technique based on a quantitative model like full maturity model is required. Many studies have been performed on strength development of the concrete using equivalent age which is based on the apparent activation energy. In this process, it considers the effect of time and temperature simultaneously. However, the previous models on the apparent activation energy of concrete with mineral admixtures have limitation, and they have not considered the effect of temperature on strength development. In this paper, the apparent activation energy with GGBFS replacement ratio was calculated through several experiments and used to predict the compressive strength of GGBFS concrete. Concrete and mortar specimens with 0.6 water/binder ratio, and 0 to 60% GGBFS replacement were prepared. The apparent activation energy (*E_a_*) was experimentally derived considering three different curing temperatures. Thermodynamic reactivity of GGBFS mixed concrete at different curing temperature was applied to evaluate the compressive strength model, and the experimental results were in good agreement with the model. The results show that when GGBFS replacement ratio was increased, there was a delay in compressive strength.

## 1. Introduction

Ground granulated blast furnace slag (GGBFS) is widely used in various engineering applications to replace the ordinary Portland cement (OPC) [1,2,3]. However, GGBFS is sensitive to curing conditions and exhibit slow strength development [4,5,6,7,8]. In order to predict the strength behavior in the concrete with GGBFS, much research have been performed based on cement hydration phenomena [9]. Moreover, it is necessary to estimate the mechanical properties of GGBFS concrete, such as compressive strength, splitting tensile strength, elastic modulus, creep, shrinkage, etc. Among the mechanical properties used in the design, compressive strength is the most important. There are different models for strength development, but among them, the maturity model is the best model. This model is based on the age concept. It has been widely used to evaluate the compressive strength of the concrete on the assumption of linear relationship with temperature [10,11,12] or a nonlinear relationship with the chemical reaction rate of the cement [13]. By considering curing temperature range and the accuracy of the prediction result, the equivalent age model is widely used for the interpretation of strength development, which incorporates the chemical reaction rate of the cement [13]. In the chemical reaction rate model, it is considered that apparent activation energy (*E_a_*) is a key parameter with the curing temperature on the hydration reaction [14,15,16,17,18,19,20]. The *E_a_* is indirectly proportional to compressive strength, as suggested in ASTM C 1074-11 [21]. The application of *E_a_* in the equivalent age model for normal concrete and the related strength prediction model is reasonably agreed with the test results. However, the concrete with GGBFS exhibits several differences compared to normal concrete in regards to the strength development attributed to a reduction of compressive strength in early age, caused by retardation of the setting. When the GGBFS comes into contact with concrete pore solution, the impermeable acid film surrounds the particles on the surface is destroyed; therefore, the chemical reaction in concrete would start. The delay in initial compressive strength of concrete with GGBFS is owing to insufficient alkalinity of cement paste [4,5,6,22,23]. In addition, Escalante et al. [4,5] studied the hydration of Portland cement with GGBFS under curing conditions. The hydration reaction was measured for six months at 10 °C, 30 °C and 50 °C curing temperature of cement pastes with 30%, 50% GGBFS replacement in 0.5 and 0.35 W/C ratio. The highest hydration reaction rate was found with 30% GGBFS at 50 °C in 0.5 W/C, while the lowest was shown by 50% GGBFS replacement at 10 °C in 0.35 W/C. Thus, a prediction of concrete compressive strength development with GGBFS by using a full maturity model by evaluating *E_a_* is required. The present study is aimed to predict the compressive strength development of the concrete with GGBFS by calculating the *E_a_*. 

## 2. Prediction of the Compressive Strength Based on Maturity Theory

### 2.1. Maturity

Maturity is a function that quantitatively expresses the effect of curing temperature and time on strength development of the concrete. Therefore, the maturity theory by considering curing temperature and time function can be defined as:

(1) $$M=\int_{0}^{t}H(T)dt,$$

where *M* and *h*(*T*) is maturity and maturity function, respectively. *T* denotes curing temperature over the age of *t*.

The maturity function is expressed in linear Equation (2) considering temperature and the age [10], whereas, the equivalent age can be calculated by Arrhenius chemical reaction rate (Equation (3)) [12,13,24].

(2) $$\sum_{0}^{t}M_{s}=\sum \left(T-T_{0}\right)\Delta t,$$

where *M_s_* is maturity at age *t. T* is the average temperature (°C) of the concrete during the time interval, and *T*_0_ is reference temperature (−10 °C) [11].

(3) $$k_{T}=A\cdot {exp}^{\frac{E_{a}}{R\cdot T}},$$

where *k_T_* is the reaction rate constant, *A* is proportionality constant, *E_a_* is apparent activation energy (kJ/mol), *R* is gas constant (8.314 J/mol∙K), and *T* is the absolute temperature (Kelvin). Thus, the equivalent age (*t_e_*) can be calculated by the following equation:

(4) $$t_{e}=\frac{\int_{0}^{t}H(T)dt}{H(T_{r})},$$

where *H*(*T*) is maturity function at different curing temperature (*t)*, whereas, *H*(*T_r_*) represents the maturity function at fixed curing temperature, i.e., 20 °C (293 K). The equivalent age means curing time at standard temperature (20 °C). By considering *E_a_*, *t_e_* can be derived as:

(5) $$te=\int_{0}^{t}{exp}^{\frac{E_{a}}{R}\cdot (\frac{1}{T_{r}}-\frac{1}{T})}dt,$$

where *T* is the average curing temperature of the concrete during a time interval and *T_r_* is the absolute or fixed temperature, i.e., 20 °C (293 K).

The strength development analysis using maturity was carried out by considering curing temperature and duration. Other researchers have suggested a hyperbolic regression model (Equation (6)) which gives more accurate predictions compared to an exponential function [24,25,26,27]. In addition, the following model was implemented by introducing a third variable to explain the dormant period during the hydration process of cement.

(6) $$S=\frac{S_{u}k_{T}(t_{e}-t_{0})}{1+k_{T}(t_{e}-t_{0})}.$$

*S* is predicted compressive strength, whereas, *S_u_* represents the obtained experimental compressive strength at 28 days of curing. *k_T_* is the reaction rate constant at curing temperature (*T*). *t_e_* and *t*_0_ represent the equivalent age and the age when compressive development starts (final setting time), respectively.

### 2.2. Reaction Rate Constant(k_T_) and Apparent Activation Energy (E_a_)

The cement reacts with water at an early age resulting hydration reaction. Due to the hydration of cement paste in concrete, it develops strength. The hydration rate is governed by the reaction rate constant of the point when the cement paste and water react [24,28]. The hydration degree of Portland cement can be derived by the weight ratio of reaction products. The weight ratio of reaction products can be determined by non-hydrated cement using an electron microscope or X-ray diffraction analysis [29,30,31,32]. Also, the degree of hydration can be measured by the amount of water, as well as heat generation, and the compressive strength in an indirect way. The most common indirect hydration measurement method used in Portland cement is the micro-hydration method using conduction calorimeter. It measures the amount of hydration heat generated at the beginning when hydration of cement starts with time and represents the ratio of calorific value of final hydration per weight of cement [33]. This method accurately shows the degree of hydration at the beginning of cement hydration, but cannot change the degree of hydration, due to change in curing process [27]. The reaction rate constant is the indicator of the initial gradient for the degree of hydration. There are many factors which affect the reaction rate constant. However, it is difficult to quantitatively predict the effect of temperature on the reaction rate constant. Therefore, the reaction rate constant can be represented by the function of curing temperature if other conditions are identical. It is known that the reaction rate constant is influenced by the types of cement, curing temperature, W/C (%), admixture, and humidity conditions etc. [9,34,35,36,37]. Thus, activation energy (*E_a_*) can help to calculate the reaction rate constant where minimum energy is required to occur the reaction. It has been reported that *E_a_* can vary owing to the nature of Portland cement, which has different hydration reaction patterns with the setting process, curing period, and cementitious components [38]. Some researchers have reported that *E_a_* is approximately 33.5 to 47.0 kJ/mol at early-age, and approximately 10 to 30 kJ/mol at long-term age [39,40,41,42,43,44,45,46,47,48,49]. Freisleben-Hansen and Pederson (FHP) [13] proposed an equation (Equation (7)) to estimate *E_a_* of OPC concrete as a function of curing temperature.

(7a) $$E_{a}=33.5+1.47(20-T_{a})\mathrm{kJ}/\mathrm{mol}(T_{a}<20\;{}^{\circ}C),$$

(7b) $$E_{a}=33.5\mathrm{kJ}/\mathrm{mol}(T_{a}\geq 20\;{}^{\circ}C),$$

where *E_a_* is apparent activation energy by Freisleben-Hansen and Pedersen with temperature parameter, *T_a_* is the average curing temperature of concrete during a time interval.

However, Carino pointed out that *E_a_* can be determined by the composition, powder level, type, amount, and admixture of the cement [25,26], and other researchers argue that the *E_a_* is changed by W/C ratio.

**Figure 1:**
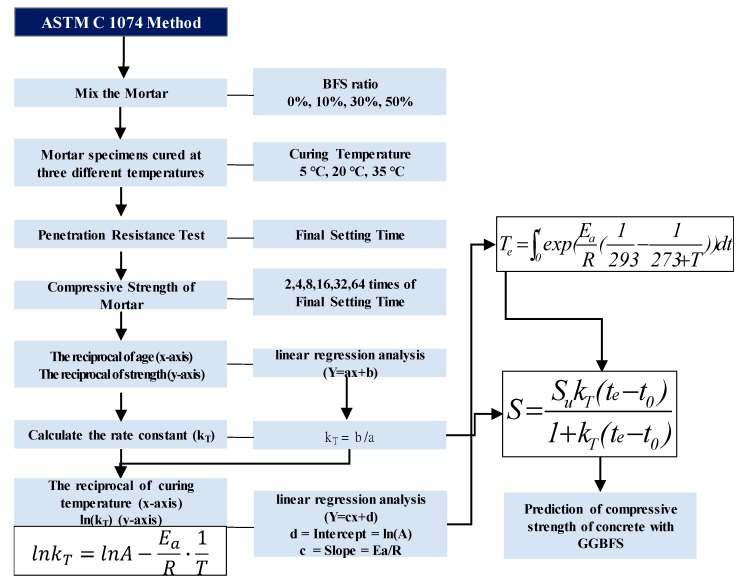
Calculation of *E_a_* and estimation of compressive strength of concrete according to ASTM C 1074.

The hydration reaction of Portland cement can be formulated with *E_a_*. Therefore, ASTM C 1074 [21] has suggested the method to determine the *E_a_*. The procedure for determining the *E_a_* and the compressive strength prediction procedure, according to ASTM C 1074 is shown in Figure 1.

The final setting time of the mortar cured at three different temperatures is measured. The compressive strength of mortar is measured at 2, 4, 8, 16, 32 and 64 times as the final setting time. By plotting the reciprocal of the age(*x*-axis) versus the compressive strength (*y*-axis), the y-intercept of the linear regression line can be obtained from the regression analysis. *E_a_* can be calculated by plotting the reciprocal of the curing temperature(*x*-axis) versus the reciprocal of *lnk_T_* (*y*-axis) and dividing the gradient of the linear regression line obtained from the regression analysis. The equivalent age can be calculated by *E_a_* of GGBFS concrete from Equation (5), and the compressive strength development is analyzed by the calculated equivalent age and *k_T_* from Equation (6).

## 3. Experimental Program

### 3.1. Experimental Variables

Experimental variables were GGBFS replacement ratio and curing temperature at 0.60 water/binder (W/B) ratio. Details of these ratios are shown in Table 1.

### 3.2. Materials

#### Cement and GGBFS

The cement used in the present study was ordinary Portland cement Type I containing 3.14 g/cm^3^ specific gravity. Blaine specific surface area of cement was 3230 cm^2^/g. GGBFS was used according to ASTM C 989 [50] with 2.84 g/cm^3^ specific gravity. Blaine specific surface area of cement was 4260 cm^2^/g. The chemical analysis of these materials are given in Table 2. The chemical composition OPC and GGBFS were carried out by X-ray fluorescence (XRF) instrument.

### 3.3. Mixture Proportions

The concrete mixture proportion is presented in Table 3. Four types of specimens with different GGBFS replacement ratio were prepared. The mortar was prepared by removing less than 5 mm coarse particle as shown the mixture proportion in Table 3 using 600 Am sieve according to ASTM C33 [51] to maintain the quality of the concrete. Different amounts of superplasticizer were used to equalize the workability of GGBFS and OPC concrete. When we have added the high amount of superplasticizer in GGBFS, the workability was reduced (results are not shown). Therefore, it is required to maintain workability. Hence, we have chosen a different amount of superplasticizer. We have taken the high amount of superplasticizer in OPC (without GGBFS) to mix the concrete properly owing to delay the setting time whereas, in case of GGBFS replacement, specimens were mixed properly even at a low amount of superplasticizer.

### 3.4. Prepared Specimens

Firstly, 0.1 m^3^ of concrete mix was prepared for each batch. Cement, GGBFS, fine aggregate and coarse aggregate were mixed in a screw type mixer for 1 min and 30 s. Thereafter, water and superplasticizer were added, and the mixture was mixed for 3 min and 30 s. In order to obtain the mortar, the fresh concrete was passed through a 5 mm sieve, and cast into a mold (200 mm × 100 mm) for penetration resistance test according to ASTM C403 [52], whereas, compressive strength of a mold (50 mm × 50 mm × 50 mm) according to ASTM C109 [53]. All specimens were cured at 5 ± 2 °C, 20 ± 2 °C, 35 ± 2 °C and 60% (±2.5%) relative humidity. The curing condition was selected according to ASTM C39 [54].

### 3.5. Test Methods

#### 3.5.1. Penetration Resistance Test for Determining Setting Time

In order to measure the mortar setting time, the penetration resistance of the mortar was measured using a standard needle at a regular time interval according to ASTM C 403 [52]. From the plot of penetration resistance versus elapsed time, the initial and final setting times were determined. For mortar with 50% GGBFS content, the setting time could not be measured under curing conditions of 5 ± 1.5 ° C. Therefore, the time when the compressive strength of the mortar reaches 4 MPa was considered as the final setting time by the alternative method proposed in ASTM C 1074 [21].

#### 3.5.2. Compressive Strength Test of Mortar and Concrete

The compressive strength of mortar was measured by 30 t class universal testing machine, and the average value was used by measuring the compressive strength in a triplicate set of specimens at 2, 4, 8, 16, 32 and 64 times for the final setting time [21]. In addition, the compressive strength of the concrete was measured 200 t class universal testing machine to measure the compressive strength in a triplicate set of specimens at ages 3, 7, 14, and 28 days, and the average value was considered as a result.

## 4. Results and Discussion

### 4.1. Setting Time of Mortars

**Figure 2:**
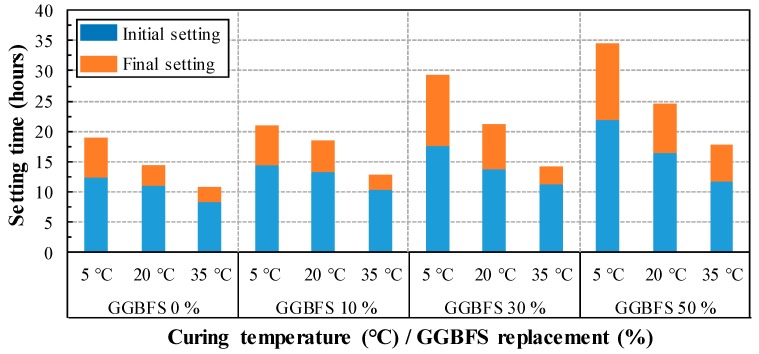
Setting time of mortar, according to curing temperature and replacement ratio of GGBFS.

The initial and final setting time of mortar with GGBFS was measured by a penetration resistance test. Figure 2 shows the effect of GGBFS and temperature on the setting time of mortar. As expected, mortars of the same GGBFS replacement ratio show a decrease in setting time at higher curing temperatures. In addition, compared with OPC mortar, as the GGBFS replacement ratio increases, the setting time increases at the same curing temperature. In case of mortar with 50% of GGBFS compared to OPC mortar at the curing temperature of 5 °C, the final setting time was delayed by 15.7 h. When mortar with 50% GGBFS was cured at 5° C and 35 °C, the final setting time was delayed by 16.9 h. The largest difference in the final setting time was 23.7 h between 50% GGBFS at 5 °C and OPC at 35 °C. It is considered that impermeable film has formed when GGBFS particle reacts with water. Due to the formation of the impermeable film results in the delay of hydration reaction, which restraining the penetration of water and ion [44,45,46,47]. Thus, this result suggests that the setting quickly occur owing to the fast reaction of hydration caused in early age at high temperature. Therefore, the setting time is affected by both curing temperature and GGBFS replacement ratio.

### 4.2. Compressive Strength of Mortars

**Figure 3:**
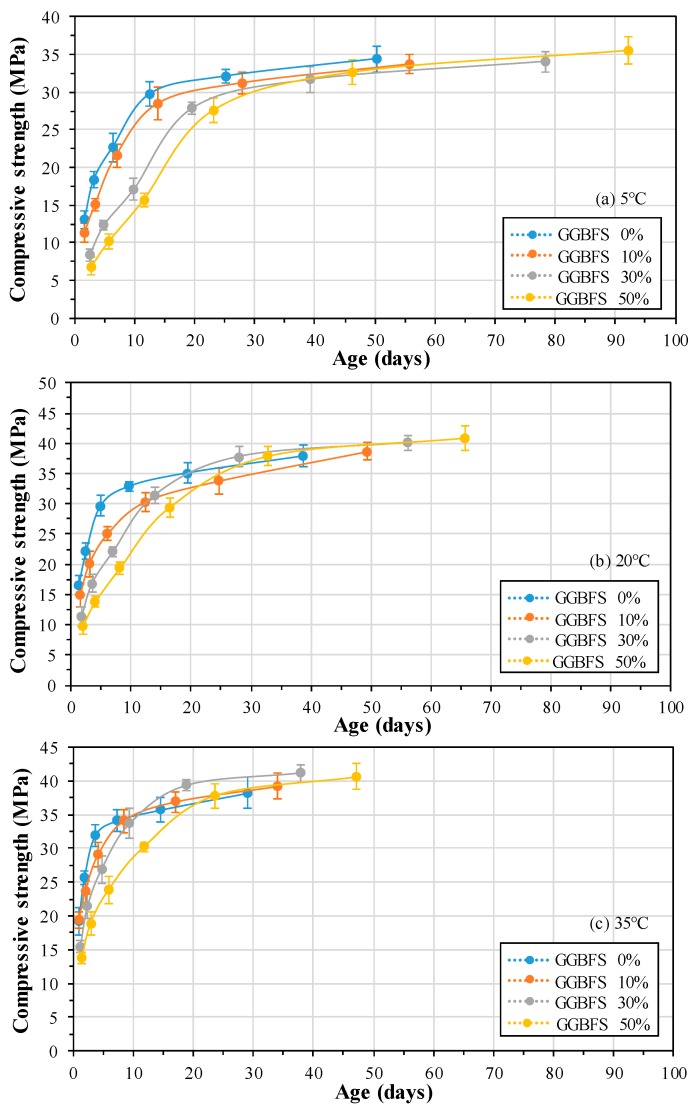
Compressive strength of mortar, according to curing temperature and replacement ratio of GGBFS (**a**) 5 °C, (**b**) 20 °C, (**c**) 35 °C.

The compressive strength of mortar with GGBFS replacement ratio at each curing temperature is shown in Figure 3. The compressive strength of mortar at an early age is found to be lowest with increasing GGBFS replacement ratio. However, with an increase in curing temperature, the compressive strength is increased. It is reported by Karim et al. that supplementary cementitious materials (SCM) in cement significantly modify the hydration kinetics and gives better performance at higher curing temperature [55]. However, the long term strength of blended cement mortar depends on curing temperature. Moreover, SCM can recover the strength once it cured in high temperature at longer duration. The early strength of higher GGBFS replacement mortar is lower, but it can be improved when it would cure at elevated temperature attributed to the quick cement hydration reaction. At 5 °C curing temperature, the compressive strength of 50% GGBFS mortar exceeded the compressive strength compared to 30% GGBFS after 39 days (Figure 3a). In addition, at 20 °C (Figure 3b) and 35 °C (Figure 3c) curing temperature, the compressive strength of 50% GGBFS mortar exceeded compare to OPC after 28 and 22 days, respectively. As the GGBFS replacement ratio increases, the crossover effect of compressive strength is delayed compared to OPC. However, the crossover effect of compressive strength decreases as the curing temperature increases. Therefore, depending on the replacement ratio of GGBFS, the hydration and degree of hydration of mortar are very sensitive to temperature.

### 4.3. Calculation of Reaction Rate Constant (k_T_) and Apparent Activation Energy (E_a_)

**Figure 4:**
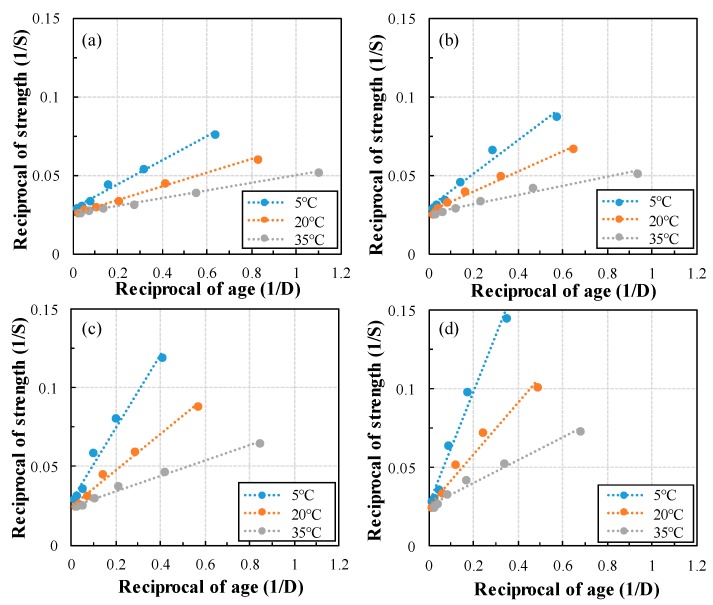
Regression analysis results for calculating *k_T_* (**a**) 0%, (**b**) 10%, (**c**) 30%, (**d**) 50%.

**Figure 5:**
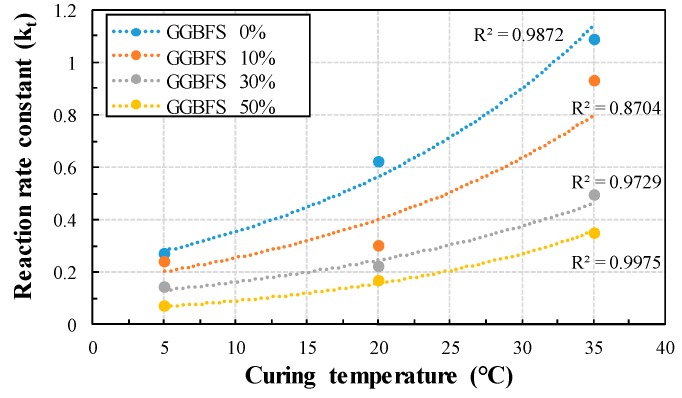
The effect of temperature on the rate constant with GGBFS replacement ratio.

The relationship between age and compressive strength was analyzed, and *k_T_* was obtained according to the GGBFS replacement ratio. Figure 4 shows the reciprocal of compressive strength versus reciprocal age. By dividing the y-intercept of the linear regression line by the slope, we can derive the *k_T_* that takes the curing temperature and the replacement ratio as variables. Figure 5 shows *k_T_* according to the GGBFS replacement ratio at each curing temperature. As the curing temperature increased, the *k_T_* increased in the form of an exponential function and showed a high correlation between 0.87 and 0.99. In addition, the lower the GGBFS replacement ratio at the same curing temperature, the higher the *k_T_*. The *k_T_* of 50% GGBFS mortar is decreased by 75% and 70% at 5 °C and 35 °C curing temperature, respectively compared to OPC. In addition, the reaction rate constant of OPC and 50% GGBFS mortar is decreased by 76% and 81% at 5 °C compared to 35 °C curing temperature, respectively. Therefore, the curing temperature and GGBFS replacement ratio have a complex effect on the *k_T_*.

**Figure 6:**
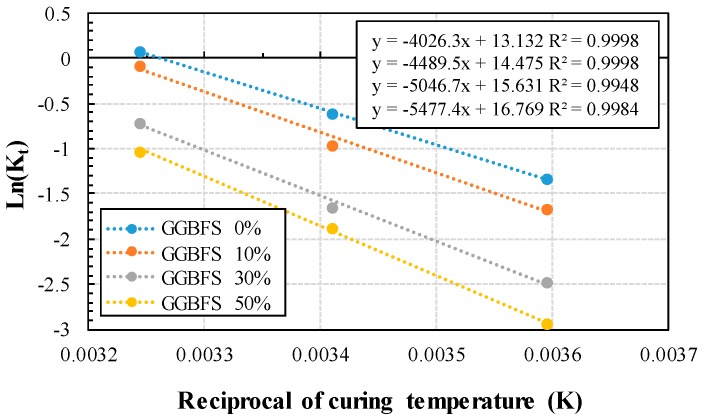
Arrhenius plot of ASTM C 1074 for calculating *E_a._*

By taking the natural logarithm of the calculated *k_T_* and plotting the reciprocal of the curing temperature (K), we can represent the Arrhenius plot, as shown in Figure 6. The gradient of the linear regression line of each GGBFS replacement ratio represents *E_a_/R*, and the y-intercept represents the value of *ln*(*A*). As the GGBFS replacement rate increases, the gradients of the linear regression line decreases to a negative value.

**Figure 7:**
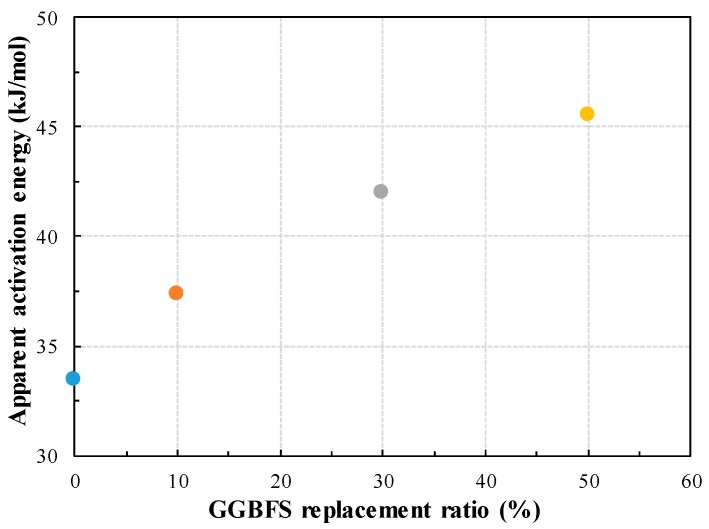
Apparent activation energy according to the GGBFS replacement ratio.

Figure 7 shows the *E_a_* results of GGBFS replacement ratio. As the GGBFS replacement ratio increases, the *E_a_* increases linearly, which considered as the result of an increase in the minimum energy for the chemical reaction of cement, GGBFS and water. For OPC mortar, *E_a_* is estimated to be 33.475 kJ/mol, which is very similar to the proposed value of Freisleben-Hansen and Pederson, i.e., 33.5 kJ/mol [13]. Wirkin et al. have found that superplasticizer has a little role on the hydration kinetics of cement and the difference in *E_a_*, with or without the superplasticizer, is insignificant, i.e., 3 kJ/mol [44]. In the present study, the *E_a_* value of 10%, 30% and 50% GGBFS is found to be 37.325 kJ/mol, 41.958 kJ/mol and 45.541 kJ/mol, respectively.

### 4.4. Prediction of Compressive Strength of Concrete with GGBFS

#### 4.4.1. Compressive Strength of Concretes

**Figure 8:**
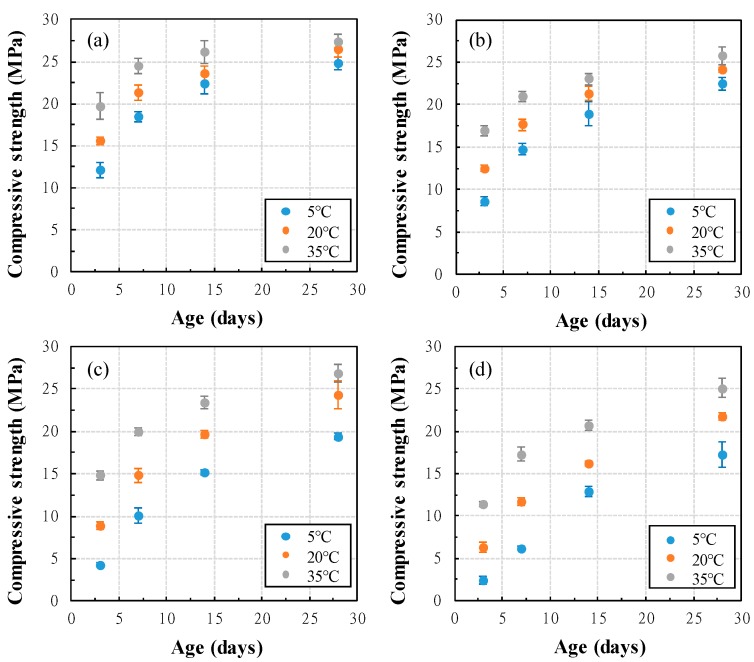
Compressive strength of concrete with curing temperature and GGBFS replacement ratio (**a**) 0%, (**b**) 10%, (**c**) 30%, (**d**) 50%.

This study measured the compressive strength of concrete with GGBFS at the age of 3, 7, 14, and 28 days and used the average of compressive strength of three specimens as a result. The results of compressive strength with varying curing temperature and GGBFS replacement ratio are shown in Figure 8. At higher curing temperatures, the compressive strength increased, and as the GGBFS replacement ratio increased, the compressive strength decreased. In addition, the difference in compressive strength with the change of curing temperature is the largest at three days of age. As the GGBFS replacement ratio increased, the difference in compressive strength, due to curing temperature increased. Especially, 28 days of age, the difference in compressive strength of OPC concrete at curing temperature of 5 °C and 35 °C was about 2.6 MPa, and the difference in compressive strength of 50% GGBFS is about 7.9 MPa. At curing temperatures of 5 °C, the compressive strengths of OPC, 10%, 30% and 50% GGBGS at three days were 12.1 MPa, 8.6 MPa, 4.3 MPa and 2.4 MPa, respectively. By increasing the GGBFS replacement ratio at low curing temperature causes delayed development of compressive strength in early age. Therefore, GGBFS concretes have different compressive strengths than OPC concrete. Thus, accurate prediction is necessary.

#### 4.4.2. Prediction of Compressive Strength of Concrete with GGBFS

**Figure 9:**
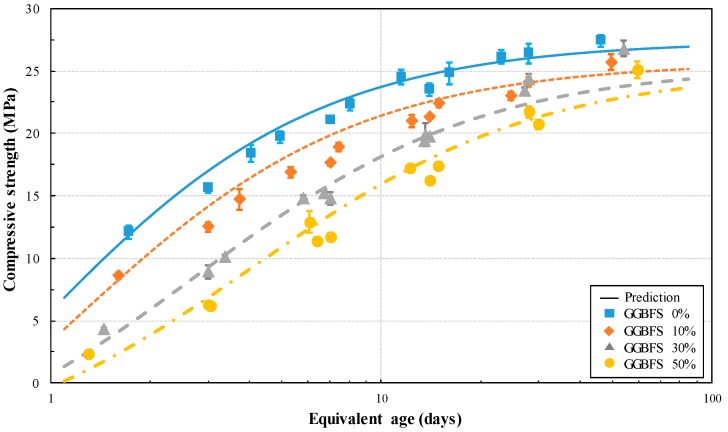
Prediction of compressive strength in concrete using GGBFS based on equivalent age.

**Figure 10:**
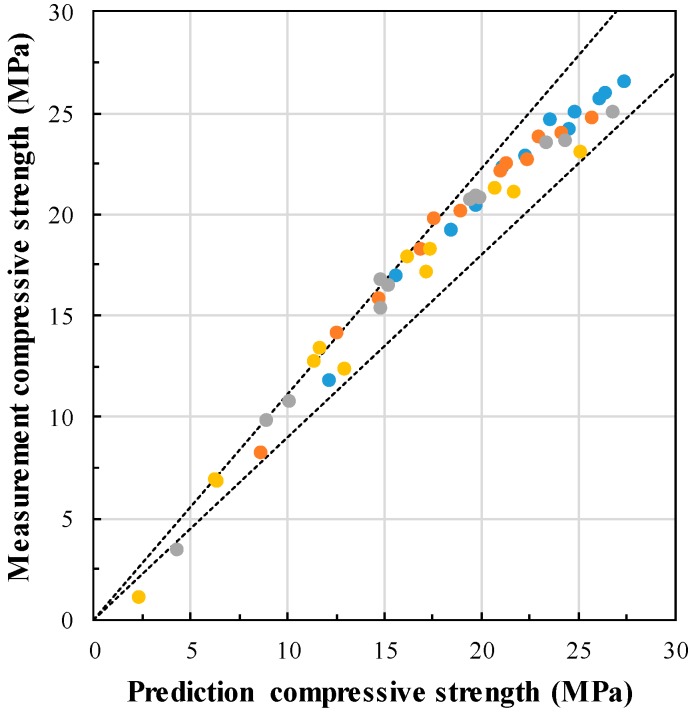
Comparison between predicted compressive strength and Measurement compressive strength.

The maturity model applied in this study is the equivalent age model, as shown in Equation (5), and the equivalent age was derived using calculated *E_a_*. The compressive strength prediction, according to the equivalent age by GGBFS replacement ratio, is shown in Figure 9. From this figure, it is found that the compressive strength at an early age is delayed as GGBFS replacement ratio increased. The compressive strength development prediction curve approximately overestimates the compressive strength, but similarly simulates the delayed expression of initial compressive strength, due to the increase of GGBFS replacement ratio. Figure 10 compares the predicted compressive strength with the measured compressive strength. The compressive strength is predicted similarly in all compressive strength regions. Thus, very high correlation (R^2^ = 0.91) is obtained. This proves that the compressive strength of concrete can be predicted in all strength at various curing temperatures and GGBFS replacement ratio.

## 5. Conclusions

From the above results and discussion, it is found that curing temperature and GGBFS replacement ratio have a significant effect on the development of concrete compressive strength. The addition of GGBFS also reduces the compressive strength of early age. As a result, the apparent activation energy and the compressive strength of concrete are affected by the GGBFS replacement ratio and curing temperature. Therefore, the existing compressive strength prediction model, i.e., Carino for OPC is not suitable for GGBFS concrete. The *E_a_* of the proposed OPC, 10%, 30% and 50% of GGBFS are found to be 33.475 kJ/mol, 37.325 kJ/mol, 41.958 kJ/mol and 45.541 kJ/mol, respectively. Therefore, the equivalent age using *E_a_* and a high level of accuracy (R^2^ = 0.91) is obtained.

## Figures and Tables

**Table 1:** The experimental variables.

Experimental Level	Items
W/B	0.60
Replacement ratio of BFS (%)	0, 10, 30, 50
Curing temperature (°C)	5, 20, 35
Fresh mortar	setting time (initial and final)
Hardened mortar	compressive strength (2, 4, 8, 16, 32, 64 times of final setting time)
Hardened concrete	compressive strength (3, 7, 14, 28 days)

**Table 2:** Chemical analysis of materials. GGBFS, ground granulated blast furnace slag.

Component	Portland Cement %	GGBFS %
SiO_2_	21.07	35.35
Al_2_O_3_	5.00	14
Fe_2_O_3_	2.92	0.36
CaO	62.40	41.91
MgO	2.07	7.74
SO_3_	2.34	0.1
K_2_O	0.59	–
Na_2_O	0.26	–
LOI	1.19	0.31
Insoluble	0.41	0.21
Cl	0.05	0.02
Free Lime	1.70	–
Total (%)	100	100

**Table 3:** Concrete mixture proportions.

W/B	GGBFS (%)	S/a (%)	Mix Composition (kg/m^3^)					
			W *	GGBFS	C *	S *	G *	SP *
0.60	0	46	217.2	0	362	798	912	1.156
	10			36.2	325.8	794	910	0.976
	30			108.6	253.4	792	908	0.659
	50			181	181	790	906	0.481

* W = water, C = cement, S = sand, G = coarse aggregates, and SP = Superplasticizer.
